# Supplementation with inulin reverses cognitive flexibility alterations and modulates the gut microbiota in high-fat-fed mice

**DOI:** 10.3389/fnbeh.2024.1445154

**Published:** 2024-11-06

**Authors:** Gabriela González-Velázquez, José Félix Aguirre-Garrido, Rigoberto Oros-Pantoja, Isidro Daniel Salinas-Velarde, Irazú Contreras, José Antonio Estrada, Alexandra Estela Soto-Piña

**Affiliations:** ^1^Facultad de Medicina, Universidad Autónoma del Estado de México, Toluca, Estado de México, Mexico; ^2^Departamento de Ciencias Ambientales, Universidad Autónoma Metropolitana-Lerma, Lerma, Estado de México, Mexico

**Keywords:** supplementation with inulin, high-fat diet, cognitive flexibility, gut microbiota, body composition

## Abstract

**Introduction:**

Alterations in cognitive performance are associated with inadequate nutritional states and diet composition. Prebiotics, such as inulin, are substances that can modulate the gut microbiome and, consequently, brain function by producing metabolites such as short-chain fatty acids (SCFAs). This study aimed to evaluate the effect of supplementation with inulin on cognitive flexibility, body composition, and gut microbiota in a murine model exposed to a high-fat (HF) diet.

**Methods:**

CD1 mice were divided into five groups: control fed a standard diet (C), high-fat diet (HF), inulin (I), high-fat diet with inulin (HFI), and manipulation control (M). Dietary supplementation was administered for 6 weeks. Cognitive flexibility was assessed using the Attentional Set-Shifting Test (AST). In addition, body composition was measured via electrical bioimpedance and adipose tissue compartments of each mouse were removed and weighed. Finally, gut microbiota metataxonomic was analyzed through metataxonomic bacterial 16S rRNA sequencing.

**Results:**

We observed that HF group required more AST trials than the C, HFI, and I groups in the compound discrimination (CD) and extra-dimensional (ED) stages. Notably, the HFI group required fewer trials than the HF group in the ED stage (*p* = 0.0187). No significant differences in overall body composition were observed between the groups. However, the percentage of gonadal and peritoneal adipose tissue was significantly higher in the HF and I groups compared to the C group. Statistically significant differences in alpha diversity for gut microbiota were observed using the Shannon, Simpson, and Chao1 indices. The I group showed a decrease in bacterial diversity compared to the HF group. While no differences were observed between groups in the phyla Bacillota and Bacteroidotes, *Clostridium* bacteria represented a lower proportion of sequences in the I group compared to the C group. Additionally, *Lactobacillus* represented a lower proportion of sequences in the HF group compared to the C and I groups.

**Discussion:**

These findings suggest that supplementation with inulin could be a useful approach to mitigate the negative effects of an HF diet on cognitive flexibility and modulate gut microbiota composition.

## 1 Introduction

Cognitive function involves learning, retaining, and recalling information (Luine, 2014). It includes executive functions, such as reasoning and problem-solving, working memory, and cognitive flexibility (Harvey, 2019; Jones and Graff-Radford, 2021). Cognitive flexibility is defined as the ability to adapt self-behavior adequately and efficiently according to a changing environment, often measured through task-switching tests (Dajani and Uddin, 2015). Neurons in the medial prefrontal cortex play a key role in cognitive control tasks, such as attentional set-shifting or attentional selection processes (Fodoulian et al., 2020; Colacicco et al., 2002), enabling set- or task-switching by encoding trial feedback information (Spellman et al., 2021).

Bidirectional interactions between the brain and the gut, involving neural, endocrine, and immune pathways, are well established (Mayer et al., 2022). The neuro-immune-endocrine axis plays an important role in maintaining systemic homeostasis. Therefore, alterations in gut function are increasingly recognized as being associated with central nervous system (CNS) disorders, with gut dysfunction often preceding the onset of neurological symptoms (Margolis et al., 2021).

The gut microbiota communicates and promotes changes in the CNS by secreting various bioactive metabolites, including short-chain fatty acids (SCFAs) and folate (Tooley, 2020). It is also known that several bacterial strains in the intestinal lumen can secrete neurotransmitters or their precursors (e.g., serotonin, noradrenaline, dopamine, and *γ*-aminobutyric acid), as well as endotoxins, which enter the circulatory system and exert regulatory effects on the CNS (Silva et al., 2020).

Diet is a key factor in shaping both the gut microbiota and brain function throughout one’s lifespan (Berding et al., 2021). Obesity, often caused by high-fat (HF) or low-quality diets, can lead to the overproduction of circulating free fatty acids, systemic low-grade inflammation (Tan and Norhaizan, 2019), changes in the profile of the intestinal microbiome, and compromised blood–brain barrier integrity, all of which are closely associated with cognitive impairment, including deficits in cognitive flexibility (Magnusson et al., 2015; Leigh and Morris, 2020).

Inulin is a polysaccharide composed of fructosyl units. It is naturally found in over 3,000 plant species, including chicory roots, dahlia tubers, bananas, wheat, garlic, leeks, onions, yacon, and Jerusalem artichoke (Wieërs et al., 2019; Fontané et al., 2018). Its widespread presence in nature makes inulin an easily accessible fiber. In addition, it is associated with various health benefits, including weight loss, modulation of depressive-like behavior (Qin et al., 2023), and stimulation of probiotic bacterial growth, which may be beneficial for the gut–brain axis, cognitive function, and neurogenesis (Szewczyk et al., 2023).

Colonic bacteria ferment prebiotic fibers such as inulin into SCFAs (Markowiak and Śliżewska, 2017). While some SCFAs are absorbed by colonocytes, those that enter systemic circulation reach other tissues, promoting health benefits through the modulation of the gut–brain axis (Thursby and Juge, 2017). An imbalance in this axis is linked to neuropathologies such as depression, where reduced SCFA levels may be associated with altered brain serotonin (5-HT) regulation (O’Riordan et al., 2022). Given that SCFAs possess immunomodulatory properties, they may be promising candidates for treating neuroinflammatory conditions (Majumdar et al., 2023). Previous studies have shown that agave inulin, in combination with probiotics, can reduce the production of proinflammatory cytokines and promote the synthesis of SCFAs (Romo-Araiza et al., 2018; Romo-Araiza et al., 2023).

Thus, this study aimed to investigate whether supplementation with inulin can enhance cognitive flexibility and whether these effects are related to changes in body composition and gut microbiota in an *in vivo* model of HF diet.

## 2 Materials and methods

The Research Ethics Committee of Facultad de Medicina approved and registered this study at Universidad Autónoma del Estado de México (CONBIOETICA-15-CEI-002-20210531). All experiments were carried out in accordance with the bioethical considerations of the Mexican Official Standard NOM-062-ZOO-1999.

### 2.1 Experimental animals

Male CD1 mice were bred in the animal facilities of Facultad de Medicina at Universidad Autónoma del Estado de México. They were maintained on a 12/12 h light/dark cycle with *ad libitum* access to food and water. Supplementation was initiated at 24 weeks of age. Mice were housed individually in separate cages.

The experimental groups were organized as follows: Control (C), High-fat (HF), Inulin (I), and High-fat/Inulin (HFI), with *n* = 6 per group, except for the C group, which had *n* = 7. The sample size for each group was determined using the resource equation method. The mice were kept in individual cages during the intervention.

### 2.2 Diet formulation and experimental procedure

Dietary supplementation was carried out for 6 weeks. The C and I groups were fed with standard laboratory food (Labdiet 5,001, rodent), and the HF and HFI groups were fed with a diet supplemented with 20 g of lard/100 g of standard diet. Inulin supplementation was administered for 6 weeks via drinking water at 40 mg/mL of blue agave inulin (Edulag, Jalisco, México).

### 2.3 Attentional set-shifting test

The Attentional Set-Shifting Test (AST) was carried out over a period of 9 days as follows:

Days 1–4, Food restriction. Food consumption was restricted to 3.5 g of food per mouse for all groups, and animals were moved to the area where the test would be applied. On day 4, the bedding was changed and remained the same until the final day of the test.

Food restriction was maintained until the final day of the test, ensuring that animals did not lose more than 20% of their body weight. To avoid additional stress for experimental animals during the test, they were familiarized with the manipulator hand, one at a time, for one minute.

Days 5–6, Acclimatization/Habituation. From this phase until the application of the test, a white plexiglass box measuring 20 cm x 51 cm x 25 cm, divided into three sections with a removable divider that separated one-third of the rest of the box, was used, placing bedding from the cage of the animal under examination.

The two other sections contained two ceramic containers, each with a treat (1/8 Honey Nut Cheerio®, Nestlé) as a reward. Animals were allowed to explore and become familiar with the test box for 3-min intervals over 1 h.

Day 7, Training. A treat was placed in each ceramic container inside the Plexiglas box. The treat was then covered with bedding from the test animal’s cage, and this process was repeated to train each animal to dig for the reward. If animals failed to demonstrate the ability to dig after 2 h, a second day of training was provided. Animals that did not learn to find the reward were excluded from the study.

Days 8–9, Testing. The test was divided into seven stages (Table 1); the first three stages were performed on day 8, and the last four were performed on day 9. A stage was considered complete when the animal succeeded in choosing the positive stimulus eight consecutive times. Stage characteristics are described as follows:

- Simple discrimination (SD): In this stage, odor was used as the stimulus and cue for reward placement. The test box contained filter paper infused with a drop of clove scent to indicate the reward location, whereas the jasmine scent was used as a negative stimulus, as it was not related to the presence of rewards. Subsequently, each container was filled with bedding, and the chosen stimulus was established as the first container in which an animal dug to look for the reward.
- Compound discrimination (CD): In this stage, odor continued to be the relevant stimulus. The same odors used in the SD stage were utilized, with clove serving as the positive stimulus. To introduce an irrelevant stimulus, the bedding from the previous stage was replaced with raphia and confetti.
- Reversal 1 (R1): In this stage, provided stimuli were the same as in the previous stage, but the negative stimulus became the positive stimulus and vice versa, relating to rewards.
- Intradimensional shift (ID): In this stage, both odors and bedding compositions were modified. Odor remained the relevant stimulus. The positive stimulus for this stage was rosemary, while the negative stimulus was cinnamon. Wood pellets and small plastic spheres were used to replace bedding.
- Reversal 2 (R2): Similar to R1, changing the negative stimulus for the positive one and vice versa, using the new stimuli as in the ID stage.
- Extradimensional shift (ED). From this stage onwards, the excavation medium became the reward-relevant stimulus. Velvet was added to the bedding as a positive stimulus, while crepe paper became the negative stimulus. Lavender and peppermint odors were added as irrelevant stimuli.
- Reversal 3 (R3): Finally, the previous stage’s negative stimulus was turned positive, and vice versa.

**Table 1:** Evaluation protocol for attentional set-shifting test.

Stage	Dimension		Combinations of stimuli	
	Relevant	Irrelevant	Positive	Negative
Simple Discrimination (SD)	Odor	Excavation medium	**Clove**/sawdust	Jasmine/sawdust
Compound Discrimination (CD)	Odor	Excavation medium	**Clove**/raphia	Jasmine/metallic confetti
Reversion 1 (R1)	Odor	Excavation medium	**Jasmine**/metallic confetti	Clove/raphia
Intradimensional shift (ID)	Odor	Excavation medium	**Rosemary**/wooden balls	Cinnamon/plastic balls
Reversion 2 (R2)	Odor	Excavation medium	**Cinnamon**/plastic balls	Rosemary/wooden balls
Extradimensional shift (ED)	Excavation medium	Odor	**Velvet**/lavender	Crepe paper/mint
Reversion 3	Excavation medium	Odor	**Crepe paper**/mint	Velvet/lavender

Representative example of the application of the attentional set-shifting test (AST) through the combinations used in the progression of the stages. It shows the use of odor as a relevant dimension in the first five stages and then the change of digging medium (bedding composition) as a relevant dimension. Positive, relevant stimuli have been marked with bold type.

### 2.4 Body composition and adipose tissue analysis

One day after the AST assessment, mice were sacrificed in a CO_2_ chamber at a rate of 2 L/min of O_2_ displacement, and body composition was immediately measured using electrical bioimpedance and an ImpediVet BIS1. According to the technical specifications of the equipment, four needle electrodes were inserted subcutaneously along each animal’s body to perform the evaluation. The percentages of total water, intracellular water, extracellular water, fat mass, fat-free mass, and body mass index (BMI) were obtained. Then, the animals were dissected to obtain fat tissue compartments (inguinal, gonadal, peritoneal, and retroperitoneal), and each fat tissue was weighed using an analytical scale.

### 2.5 Metataxonomic analysis of gut microbiota

An additional manipulation control group (M) comprising five mice was added for this part of the project. The M group was not subjected to the cognitive test and the pre-test food restriction. This group was kept in the same conditions as the previous groups and fed the standard diet for the full 6-week supplementation period. In each animal, dissection of the small and large intestines was performed, and the intestinal lumens were rinsed with distilled water using a syringe filled with tamponed PBS 1X. The intestinal liquid was collected in 10 mL tubes and stored at −70°C. DNA from intestinal content samples was extracted using a Quick-DNA™ Fecal/Soil Microbe Miniprep Kit (D6010, ZYMO Research), following the instructions provided by the manufacturer. The 16S rRNA gene was amplified by PCR to confirm the presence of bacterial DNA in the intestinal content. Extracted DNA was sequenced at the Integrated Microbiome Resource (IMR) at Dalhousie University in Canada, using the V3-V4 hypervariable regions for bacteria in the samples using the Illumina MiSeq system. Bioinformatics analysis was performed using the bioinformatics server at the Microbiology and Environmental Biotechnology Laboratory at the Universidad Autónoma Metropolitana-Lerma, with the bioinformatics data processing software mothur v.1.48.0 for Linux (Schloss et al., 2009). Subsequently, to remove chimeric sequences, we used the VSEARCH source (Rognes et al., 2016).

### 2.6 Statistical analysis

All data are presented as mean ± standard error. AST results were analyzed using a two-way analysis of variance (ANOVA). Body composition, adipose tissue, and body weight data were analyzed using one-way ANOVA, followed by Tukey’s and Dunnett’s *post-hoc* tests. Analyses were conducted using GraphPad Prism version 10.2.1 software. Bacterial composition and the alpha diversity index of gut microbiota, obtained from bioinformatic analysis, were examined using one-way ANOVA and Tuckey-Kramer *post-hoc* tests. A *p*-value of ≤0.05, with a 95% confidence interval, was considered significant. These analyses were conducted using STAMP v2.1.3 software (Parks et al., 2014).

## 3 Results

### 3.1 Effects of HF diet and inulin supplementation on attentional set-shifting test performance

**Figure 1:**
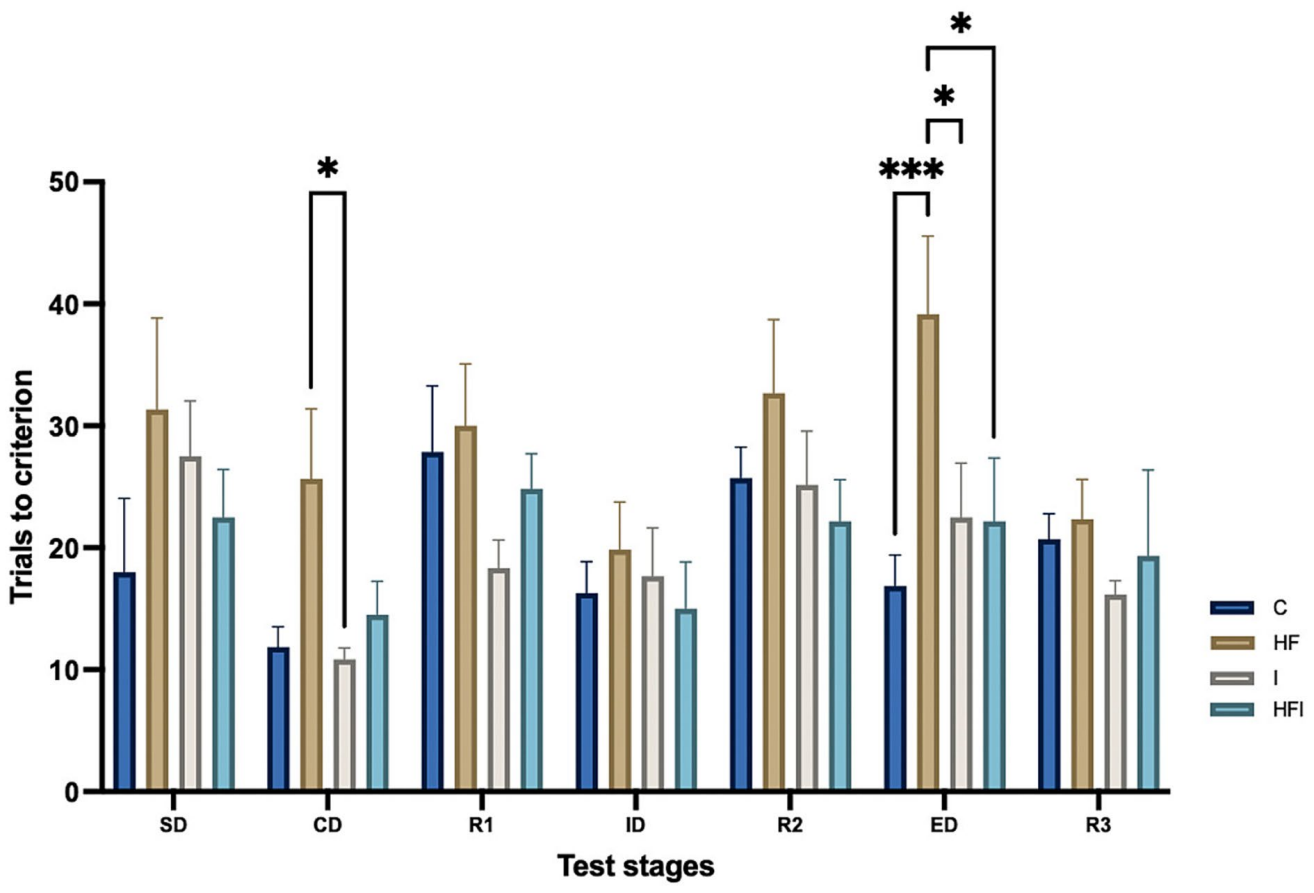
Effects of 6-week exposure to high-fat diet and inulin supplementation, plotted with standard error of the mean according to the number of attempts for Control: C, High-fat: HF, Inulin: I, and High-fat/Inulin: HFI groups, for Simple discrimination: SD, Compound discrimination: CD, Reversal 1: R1, Intradimensional shift: ID, Reversal 2: R2, Extradimensional shift: ED and Reversal 3: R3 AST stages. See individual data at ST1 in the Supplementary material.

To determine whether dietary composition and supplementation with inulin affected cognitive flexibility, we compared the AST results obtained from the experimental groups under study (Figure 1; Supplementary Table S1). Statistical analysis revealed differences between all groups (*F*_3, 147_ = 7.357, *p* = 0.0001). We also observed significant differences across the AST stages (*F*_6, 147_ = 4.193, *p* = 0.0006). However, no significant differences were found by Group × Stage interactions (*F*_18, 147_ = 0.8400, *p* = 0.65).

In the SD stage, there were no significant differences in the number of trials among groups, while in the CD stage, the HF group performed a higher number of trials to achieve completion than the I group (*p* = 0.0473). No significant differences were observed in the ID stage between experimental groups. Furthermore, there were no differences in the number of trials in any of the reversal stages (R1, R2, R3) among the C, HF, I, and HFI groups. In the ED stage, the HF group performed the highest number of trials to achieve completion, compared to the C group (*p* = 0.0008), the HFI group (*p* = 0.0187), and the I group (*p* = 0.0217) (Figure 1).

### 3.2 Effects of HF diet and inulin supplementation on body composition and body fat distribution

**Figure 2:**
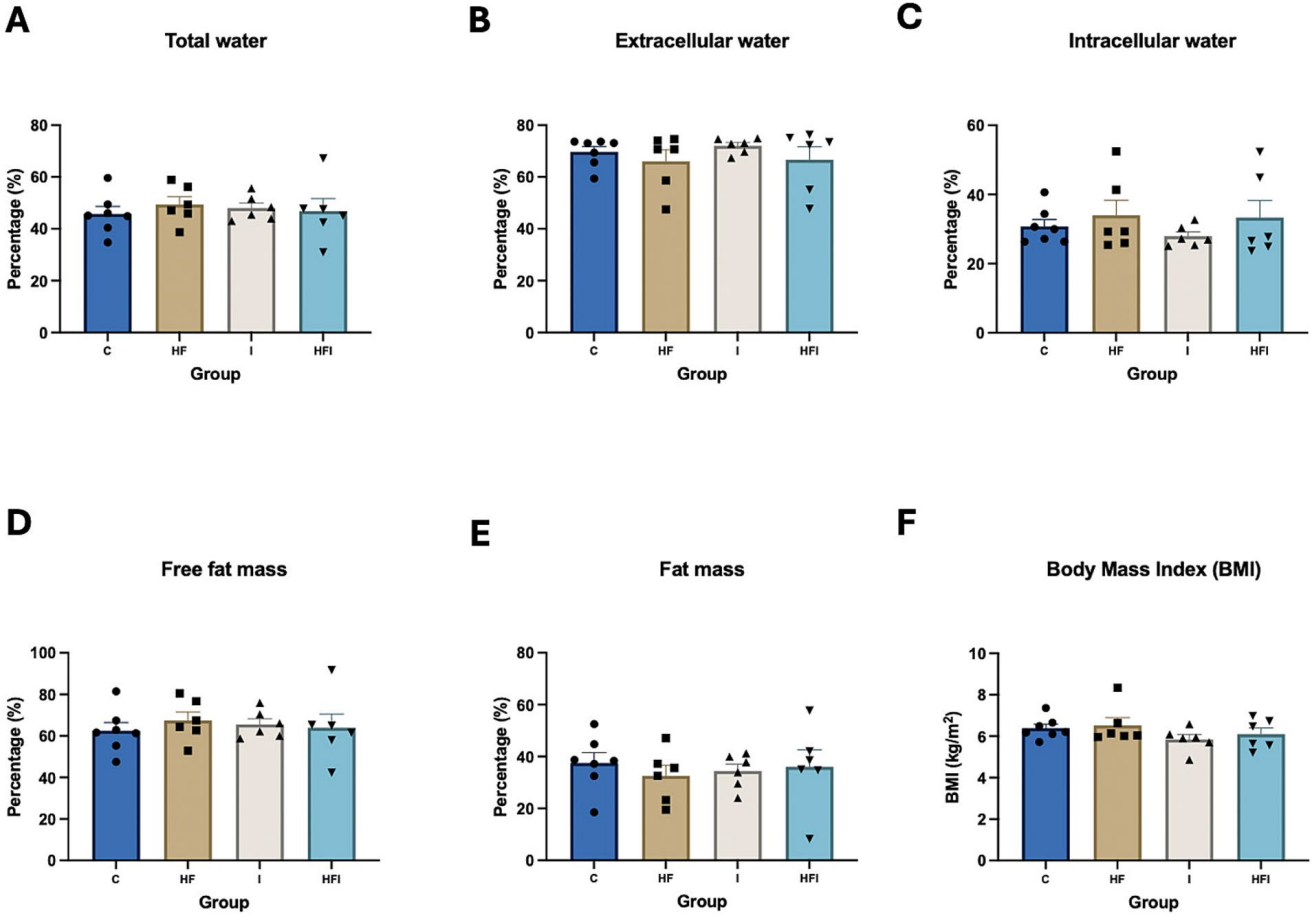
Effects of 6-week exposure to high-fat diet and inulin supplementation on body composition, represented by % total water (A), % extracellular water (B), % intracellular water (C), % fat-free mass (D), % fat mass (E), as well as body mass index (BMI) (F), among Control: C, High-fat: HF, Inulin: I, and High-fat/Inulin: HFI groups. See individual data and statistics at ST2 in the Supplementary material.

After 6 weeks of dietary intervention and the application of the AST, we measured and compared the body composition of experimental groups to evaluate the effects of HF and supplementation with inulin in our model (Supplementary Table S2). There were no significant differences in the amounts of total water (*F*_3,21_ = 0.2281, *p* = 0.8758), intracellular water (*F*_3,21_ = 0.6292, *p* = 0.6043), extracellular water (*F*_3,21_ = 0.6476, *p* = 0.5932), fat mass (*F*_3,21_ = 0.2282, *p* = 0.8757), fat-free mass (*F*_3,21_ = 0.2282, *p* = 0.8757), or BMI (*F*_3,21_ = 1.156, *p* = 0.3499) among the HF, C, I and HFI groups (Figure 2). We also measured and compared fat distribution in mice (Supplementary Table S3).

**Figure 3:**
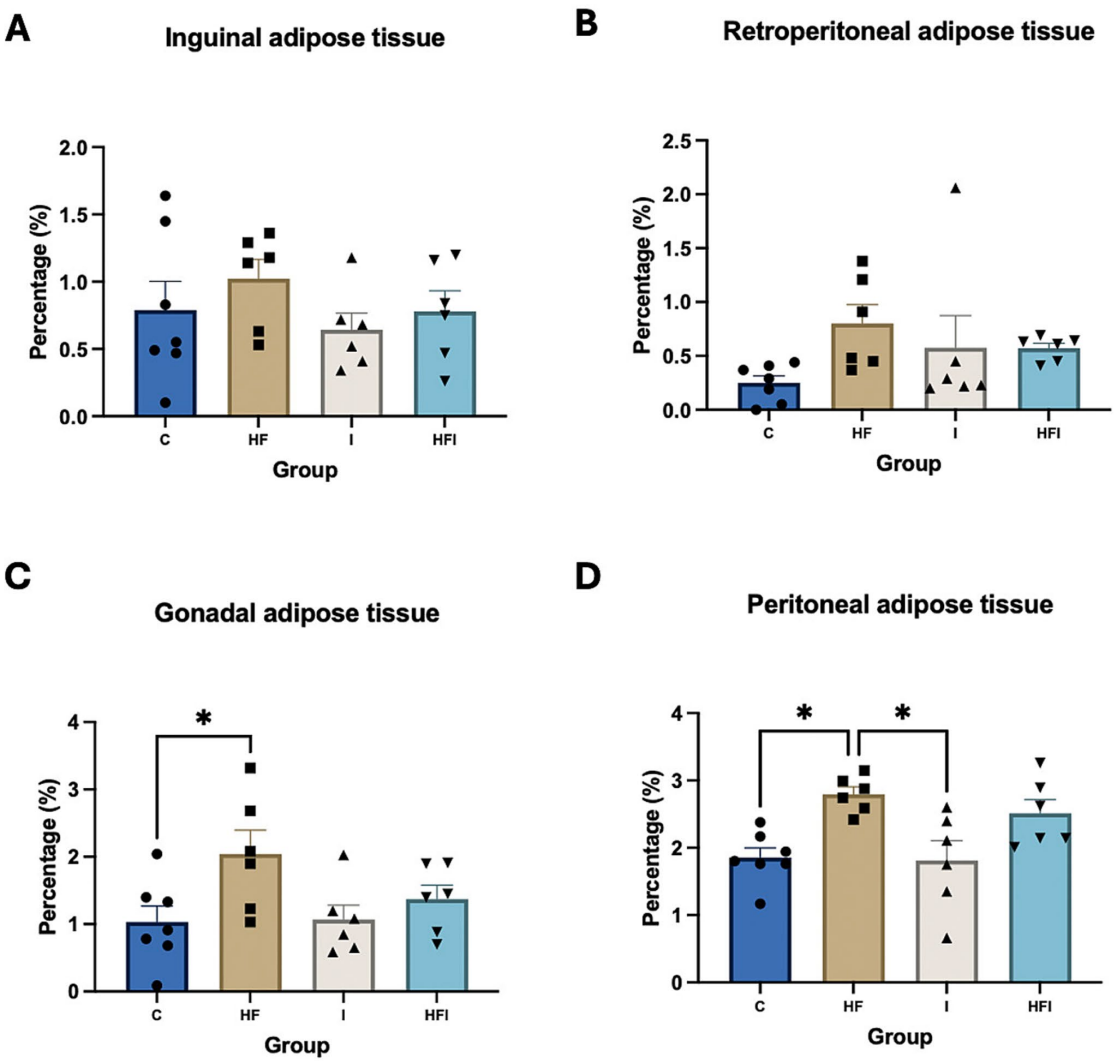
Effects of 6-week exposure to high-fat diet and inulin supplementation on body fat distribution, represented by % inguinal adipose tissue (A), % retroperitoneal adipose tissue (B), % gonadal adipose tissue (C), and % peritoneal adipose tissue (D) for Control: C, High-fat: HF, Inulin: I, and High-fat/Inulin: HFI groups. See individual data and statistics at ST3 and ST4 in the Supplementary material.

There were no significant differences in the percentage of inguinal adipose tissue (*F*_3,21_ = 0.8558, *p* = 0.4792, Figure 3A) and retroperitoneal adipose tissue (*F*_3,21_ = 1.826, *p* = 0.1734, Figure 3B). However, significant differences were observed in the percentages of gonadal adipose tissue (*F*_3,21_ = 3.23, *p* = 0.04, Figure 3C) and peritoneal adipose tissue (*F*_3,21_ = 6.10, *p* = 0.003, Figure 3D) across the different groups (Supplementary Table S4). The percentage of gonadal tissue was higher in the HF group compared to the C group (*p* = 0.0481). Moreover, the percentage of peritoneal adipose tissue was higher in the HF group compared to both the C (*p* = 0.0121) and I (*p* = 0.0115) groups.

### 3.3 Effects of HF diet and inulin supplementation on gut microbiota composition and diversity

The relative abundance of bacterial DNA and the differences presented at various taxonomic levels in intestinal contents were assessed using 16S rRNA sequencing. Significant differences in bacterial diversity were observed between groups using the Simpson, Shannon, and Chao1 diversity indexes (Table 2). *Post-hoc* tests for the Simpson index revealed that the HF group showed increased bacterial diversity compared to the I and C groups. Interestingly, a trend to a statistical difference between HF and HFI was seen (*p* = 0.07). The M group did not differ significantly from the HF and I groups.

**Table 2:** Alpha diversity of the gut microbiota of the colon of CD1 mice.

Index	Groups					ANOVA	
	M	C	HF	I	HFI	F_(DF)_	*p* value
	ab	a	b	a	ab		
Simpson	33.9 ± 4.6	18.4 ± 2.6	41.4 ± 6.2	18.1 ± 2.7	21.4 ± 7.7	4.2_(4)_	0.009
	a	ab	a	b	ab		
Shannon	4.7 ± 0.07	4.1 ± 0.09	4.6 ± 0.16	3.7 ± 0.18	3.9 ± 0.32	4.73_(4)_	0.005
	a	ab	ab	b	b		
Chao1	1,695 ± 92	1,165 ± 82	1,361 ± 177	954 ± 76	1,108 ± 165	4.56_(4)_	0.006

Alpha diversity analyzed with the Simpson, Shannon, and Chao1 diversity indexes expressed as means ± standard error of the mean for gut microbiota in the colon of CD1 mice from Control without food restriction: M, Control with food restriction: C, High-fat: HF, Inulin: I, and High-fat/Inulin: HFI groups.

Regarding the Shannon index, the I group showed a decrease in bacterial diversity compared to the HF and M groups. No significant differences were observed in the I group compared to the HFI and M groups. *Post-hoc* tests for the Chao1 index revealed a decrease in bacterial richness in the I and HFI groups vs. the M group. No significant differences were found between the I and HFI groups and the C and HF groups.

**Figure 4:**
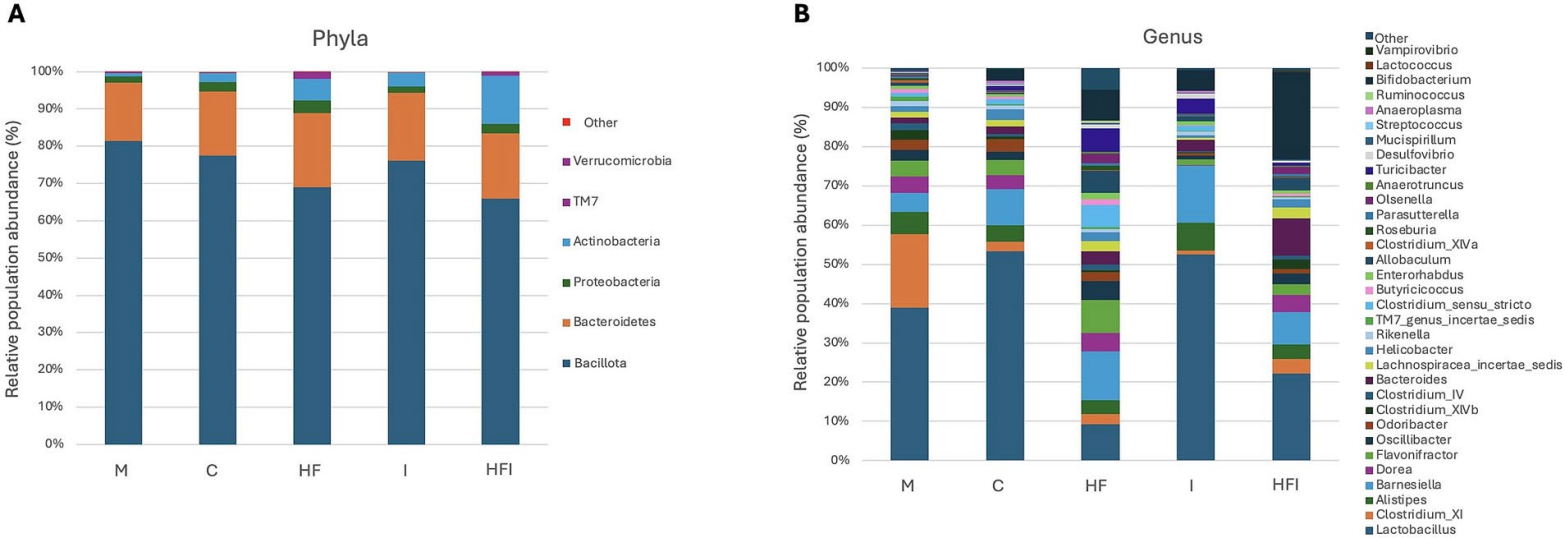
Average relative abundance (expressed in %) of phylum (A) and genus (B) taxonomic levels for bacterial gut microbiota contained in the colon of CD1 mice belonging to Control without food restriction: M, Control with food restriction: C, High-fat: HF, Inulin: I, and High-fat/Inulin: HFI groups.

Figure 4A shows the relative abundance of Bacillota (66–81.4%), Bacteroidetes (15.6–19.9%), and Actinobacteria (0.9–13.0%) as the dominant phyla across the HF, I, and HFI groups. However, no differences in the Bacillota (*F*_4,15_ = 2.619, *p* = 0.0779), Bacteroidetes (*F*_4,16_ = 0.3, *p* = 0.8736), Actinobacteria (*F*_4,10_ = 2.576, *p* = 0.1037) phylum were found between groups. The dominant genus (Figure 4B) across the C, M, I, and HFI groups was *Lactobacillus* (53.3–21.8%). In comparison, the HF group presented a 9.2% abundance of *Lactobacillus,* and the dominant genus of the HF group was *Barnesiella* (12.5%).

**Figure 5:**
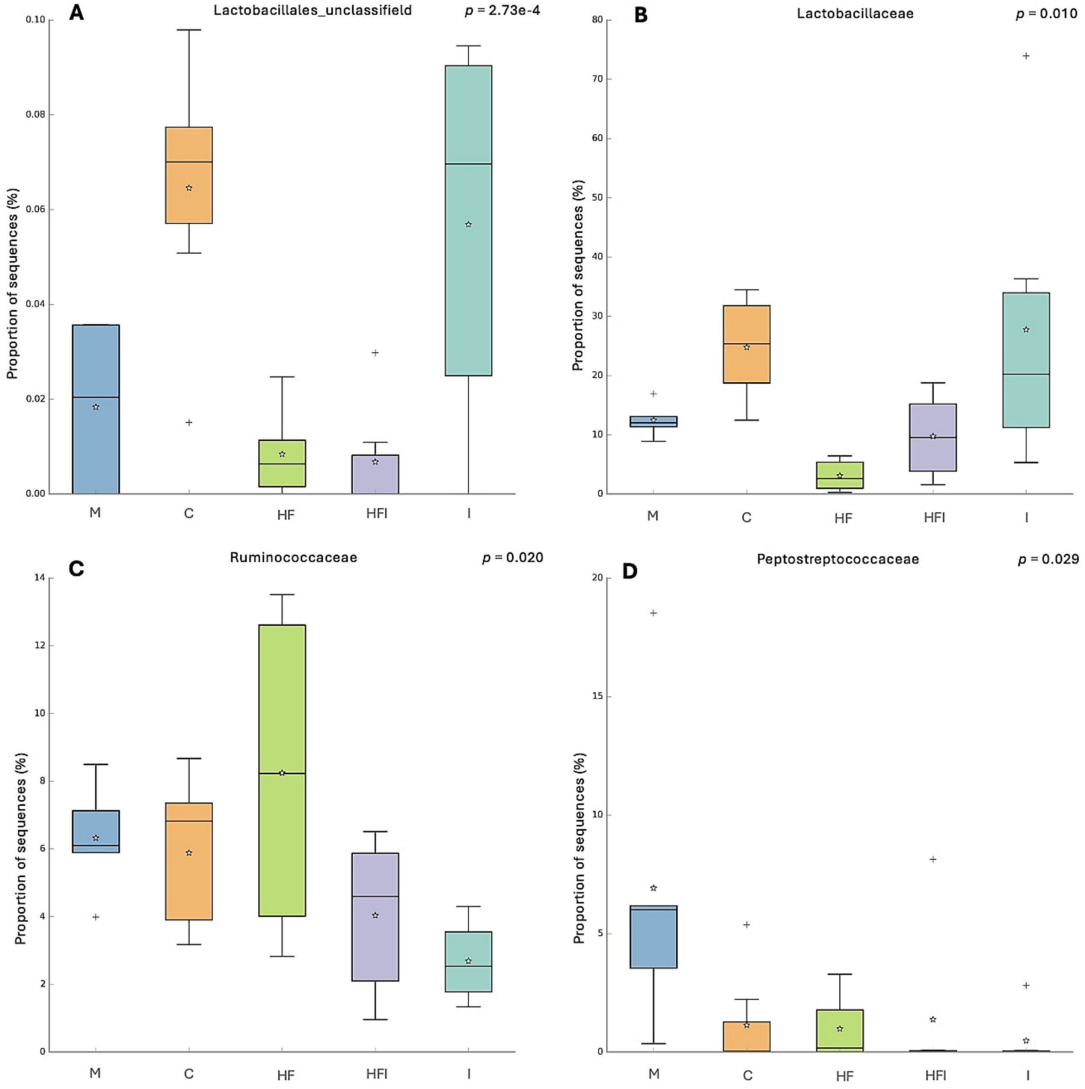
The proportion of sequences (expressed in %) of the bacterial families Lactobacillales_unclassifield (A), Lactobacillaceae (B), Ruminococcoccaceae (C) and Peptostreptococcaceae (D) present in the gut microbiota of the colon of CD1 mice from Control without food restriction: M, Control with food restriction: C, High-fat: HF, Inulin: I, and High-fat/Inulin: HFI groups. Meaning of symbols: Cross, star, and horizontal line inside the box denoted for outliner, mean, and median of the percentage of the sequences obtained.

At the family level (Figure 5), statistical differences in the proportion of sequences were found. A higher proportion of Lactobacillales_unclassified (Figure 5A) sequences were observed in the C group compared to HFI (*p* < 0.01), HF (*p* < 0.01), and M groups (*p* < 0.05). In addition, mice belonging to the I group presented a higher proportion of sequences of Lactobacillales_unclassified compared to the HF (*p* < 0.02) and HFI (*p* < 0.02) groups. Lactobacillaceae (Figure 5B) presented a lower proportion of sequences in the HF group compared to the C (*p* < 0.05) and I groups (*p* < 0.02). Ruminococcoccaceae (Figure 5C) presented a higher proportion of sequences in the HF group vs. the I group (*p* < 0.02). Peptostreptococcaceae (Figure 5D) presented a lower proportion of sequences in the I group vs. the M group (*p* < 0.05).

**Figure 6:**
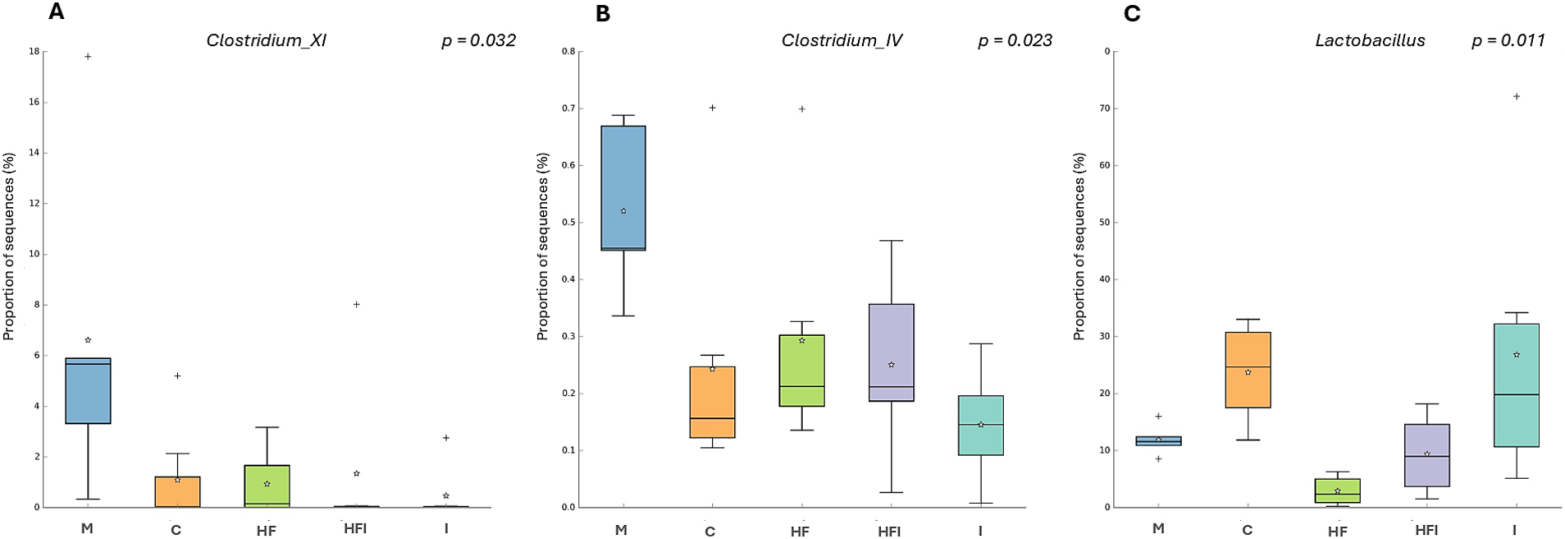
The proportion of sequences (expressed in %) of the bacterial genera *Clostridium_XI* (A), *Clostridium_IV* (B), and *Lactobacillus* (C) present in the gut microbiota of the colon of CD1 mice compared from Manipulation Control: M, Control with food restriction: C, High-fat: HF, Inulin: I, and High-fat/Inulin: HFI groups. Meaning of symbols: Cross, star, and horizontal line inside the box denoted for outliner, mean, and median of the percentage of the sequences obtained.

At the genus level, *Clostridium_XI* (Figure 6A) presented a lower proportion of sequences in the I group vs. the M group (*p* < 0.05), as did *Clostridium_IV* (Figure 6B), which presented a lower proportion of sequences in the I group vs. the M group (*p* < 0.02). In addition, *Lactobacillus* (Figure 6C) presented a lower proportion of sequences in the HF group compared to the C (*p* < 0.05) and I (*p* < 0.02) groups.

## 4 Discussion

HF diets not only contribute to the development of obesity but also negatively affect cognitive function. HF diets have been associated with decreased cognitive performance and cognitive impairment by promoting neuronal apoptosis, neuroinflammation, and neuronal insulin resistance, ultimately leading to the pathogenesis of neurological disorders (Lee et al., 2018; Kothari et al., 2017; Sah et al., 2017; Morrison et al., 2010; Zuloaga et al., 2016; Pistell et al., 2010; Liu et al., 2014; Salinero et al., 2020; Herculano et al., 2013; Gainey et al., 2016; Lin et al., 2016; Wang et al., 2018; Janssen et al., 2016; Kim et al., 2016; Marwitz et al., 2015). In this study, we observed that a 6-week HF dietary regimen impaired attention set-shifting abilities in experimental animals, particularly at both the CD and ED stages, compared to the I, HFI, and C groups. Additionally, we found that the cognitive performance of the HFI mice was similar to that of the I and C groups.

The set-shifting stages rely on fundamental cognitive functions, such as forming associations between stimuli, responses, and outcomes, as well as accurately detecting errors. These functions are primarily controlled by the prefrontal cortex (PFC) and involve key neurotransmitters such as noradrenaline (NA) and dopamine (DA) (Bissonette et al., 2013). For example, rodents exposed to chronic stress or intermittent cold stress exhibit impaired performance at the CD stage (Bondi et al., 2010; Lapiz-Bluhm et al., 2009), and this impairment can be reversed through chronic treatment with atipamezole, an adrenergic antagonist (Bondi et al., 2010).

Furthermore, NA and DA neurotransmission in the PFC is closely linked to working memory performance during the ED stage, where successful task completion is associated with phasic NA activity and tonic DA activity to maintain attentional focus (Pajkossy et al., 2018). Given this, supplementation with inulin may modulate NA neurotransmission through SCFA activity, thereby preventing cognitive impairment. Future research is needed to explore whether supplementation with inulin induces brain modifications, such as changes in SCFA receptors, neurotrophic factors, or brain plasticity-related proteins, in regions associated with cognitive function (e.g., the orbitofrontal cortex, prefrontal cortex, and hippocampus).

Studies have reported that prebiotics such as inulin may protect against the effects caused by an HF diet, reducing weight gain, adiposity, and signs of metabolic syndrome (Zou et al., 2018; Wu et al., 2022). However, we did not observe the expected effects on body weight and body composition in mice supplemented with inulin and exposed to an HF diet, which could be related to the intervention time. Consuming inulin after 18 weeks of HF diet can lead to positive health results, such as increased energy expenditure, reduced inflammation, and improved intestinal mucosal integrity (Jangra et al., 2019). In addition, the consumption of inulin may be a good option to remodel the structure and composition of the gut microbiota (Huang Y. et al., 2023).

The supplementation with inulin may also provide benefits in glucose metabolism. For instance, 16 weeks of inulin supplementation has been shown to improve glucose tolerance and insulin sensitivity while also reducing body weight in animals on an HF diet (Huang S. et al., 2023).

Moreover, combining various plant fibers may enhance the benefits of inulin. For example, the consumption of rhubarb has shown greater efficacy in reducing body weight when combined with inulin than inulin alone (Régnier et al., 2023). Similarly, supplementation with isoquercetin, a dietary flavonoid, in combination with inulin, has been shown to attenuate weight gain, reduce adipocyte hypertrophy and hepatic lipid accumulation, and improve glucose tolerance and insulin sensitivity (Tan et al., 2018). These findings have not been replicated with supplementation with inulin alone, which is consistent with our results. Therefore, the use of inulin in combination with other prebiotics or probiotics may be more effective in modulating cognitive and metabolic functions.

Regarding adipose tissue distribution, our results suggest that an HF diet influences the distribution of adipose tissue across the various assessed depots, as differences were observed in the percentages of gonadal and peritoneal adipose tissues but not in the inguinal and retroperitoneal depots. The HF diet increased the amount of fat in the gonadal and peritoneal compared to the C group. The abdominal adipose tissue in rodents, which includes gonadal and peritoneal tissue (Bloor and Symonds, 2014), is related to metabolic disorders and cardiovascular diseases (Snijder et al., 2006). HF diet-induced obesity increases the amount of gonadal white adipose and the levels of exosomal miR-222 in this region, which is implied in obesity-related insulin resistance (Li et al., 2020). In light of this, future research should explore the role of the different adipose tissue depots and the white/brown adipose tissue ratio in metabolic and cognitive diseases and how prebiotic-supplemented diets alter these depots and ratios.

As mentioned above, supplementation with inulin could reverse the increase in adipose tissue due to HF diet exposure (Zou et al., 2018). This could be because long-term inulin consumption could modulate lipolysis, adipogenesis, and adipokine synthesis by SCFAs through effects on gut hormones, the brain, and the liver (May and den Hartigh, 2021). SCFAs stimulate the release of GLP-1, PYY, and ghrelin, which, in turn, communicate directly with the brain to regulate energy intake and appetite (May and den Hartigh, 2023). However, the HFI group did not show this effect on adipose tissue in any of the regions assessed. A longer period of supplementation or an increase in inulin dose is required to observe the reported effect of inulin on the adipose tissue percentage.

We report that both consumption of an HF diet and supplementation with inulin can modify the intestinal microbiota, which may have an impact on the gut–brain axis. The supplementation with inulin can improve the richness (Li et al., 2023) and diversity (Feng et al., 2021) of the gut microbiota during the intake of the HF diet. However, we did not observe this effect in our study. According to the Chao1 index, no differences in bacterial richness were observed between the I and HF groups. Regarding bacterial diversity, the Shannon and Simpson indices showed lower diversity in the I group compared to the HF group.

In terms of relative abundance, the Bacillota, Bacteroidetes, and Actinobacteria phyla were dominant in the gut microbiota of the HF, I, and HFI groups, as previously reported (Feng et al., 2021). Moreover, inulin consumption is known to increase the proportion of Bacteroidetes, which is associated with enhanced SCFA production, and to decrease Firmicutes (now renamed as Bacillota) (Huang Y. et al., 2023). However, in this study, we did not observe a significant difference in the proportions of Bacillota and Bacteroidetes.

A previous report shows that inulin consumption above 5 g/kg/day tends to elevate the relative abundance of the Lactobacillaceae family (Zhu et al., 2017), which has been associated with cognitive function improvement and hippocampal neuroinflammation reduction (Wu et al., 2023). Our results confirm these facts since we observed that the I group showed a higher proportion of sequences in the Lactobacillaceae family and an improvement in the ED stage compared with the HF group.

The supplementation with inulin also tends to increase the relative abundance of the genera *Barnesiella Lactobacillus* and to decrease the *Clostridium XIVa* genus in mice fed an HF diet and could contribute to the regulation of intestinal SCFA production in mice fed an HF diet (Chunchai et al., 2020). This agrees with our results since our interventions modified the relative abundance of the *Clostridium XI*, *Clostridium IV,* and *Lactobacillus* genera in the experimental groups. For the latter genus, we observed that the proportion of sequences in the HF group was smaller than in the C and I groups. The SCFAs derived from bacterial fermentation, such as the Lactobacillus genus, participate in the maintenance of blood–brain barrier integrity, helping to control the passage of molecules and nutrients from the circulation into the brain, playing a central role in brain development and preservation of CNS homeostasis (Berding et al., 2021).

Therefore, regarding the gut–brain axis, it is important to analyze the impact of supplementation with inulin on cognitive function. Inulin consumption is related to the positive remodeling of gut microbiome-metabolome matrices, which can be attributed to the increase in SCFAs and the reduction in branched-chain amino acid levels, helping to decrease neuroinflammation (Kadyan et al., 2024; Hoffman et al., 2019). Therefore, chronic consumption of inulin may be considered a potential therapeutic aid for neuroinflammatory diseases and improved brain function (Boehme et al., 2020).

Although an animal model was used in this experiment, certain limitations remain, such as the inability to fully control variables such as a germ-free environment and individual variability. Murine models are valuable for studying strategies to improve human health. Future studies could focus on evaluating a longer period of dietary intervention to further corroborate and expand upon the results presented in this study.

In conclusion, prolonged consumption of high-fat diets impairs performance at the ED shift stage of the AST in mice. Notably, our study shows that supplementation with inulin can mitigate some of the negative effects of HF diets on cognitive flexibility, specifically at the ED stage of the AST. Furthermore, both inulin and HF diets have the potential to modify the gut microbiota. Therefore, supplementation with inulin may serve as a potential intervention to prevent or reverse the effects of long-term high-fat diet exposure, due to its accessibility and low cost. However, long-term supplementation with inulin could be recommended to fully achieve the cognitive and gut health benefits.

## Data Availability

The raw data supporting the conclusions of this article will be made available by the authors, without undue reservation.

## References

[ref1] Berding K. Vlckova K. Marx W. Schellekens H. Stanton C. Clarke G.. (2021). Diet and the microbiota-gut-brain axis: sowing the seeds of good mental health. Adv. Nutr. 12, 1239–1285. doi: 10.1093/advances/nmaa181, PMID: 33693453 PMC8321864

[ref2] Bissonette G. B. Powell E. M. Roesch M. R. (2013). Neural structures underlying set-shifting: roles of medial prefrontal cortex and anterior cingulate cortex. Behav. Brain Res. 250, 91–101. doi: 10.1016/j.bbr.2013.04.037, PMID: 23664821 PMC3708542

[ref3] Bloor I. D. Symonds M. E. (2014). Sexual dimorphism in white and brown adipose tissue with obesity and inflammation. Horm. Behav. 66, 95–103. doi: 10.1016/j.yhbeh.2014.02.007, PMID: 24589990

[ref4] Boehme M. van de Wouw M. Bastiaanssen T. F. S. Olavarría-Ramírez L. Lyons K. Fouhy F.. (2020). Mid-life microbiota crises: middle age is associated with pervasive neuroimmune alterations that are reversed by targeting the gut microbiome. Mol. Psychiatry 25, 2567–2583. doi: 10.1038/s41380-019-0425-1, PMID: 31092898

[ref5] Bondi C. O. Jett J. D. Morilak D. A. (2010). Beneficial effects of desipramine on cognitive function of chronically stressed rats are mediated by α1-adrenergic receptors in medial prefrontal cortex. Progress Neuro-Psychopharmacol. Biol. Psychiatry 34, 913–923. doi: 10.1016/j.pnpbp.2010.04.016, PMID: 20417676 PMC2910206

[ref6] Chunchai T. Keawtep P. Arinno A. Saiyasit N. Prus D. Apaijai N.. (2020). A combination of an antioxidant with a prebiotic exerts greater efficacy than either as a monotherapy on cognitive improvement in castrated-obese male rats. Metab Brain Dis. 35, 1263–1278. doi: 10.1007/s11011-020-00603-5, PMID: 32676884

[ref7] Colacicco G. Welzl H. Lipp H.-P. Würbel H. (2002). Attentional set-shifting in mice: modification of a rat paradigm, and evidence for strain-dependent variation. Behav. Brain Res. 132, 95–102. doi: 10.1016/s0166-4328(01)00391-6, PMID: 11853862

[ref8] Dajani D. R. Uddin L. Q. (2015). Demystifying cognitive flexibility: implications for clinical and developmental neuroscience. Trends Neurosci. 38, 571–578. doi: 10.1016/j.tins.2015.07.003, PMID: 26343956 PMC5414037

[ref9] Feng Y. Feng J. Wang L. Meng A. Wei S. Cui J.. (2021). Short-chain inulin modulates the cecal microbiota structure of leptin knockout mice in high-fat diet. Front Microbiol. 12:12. doi: 10.3389/fmicb.2021.703929, PMID: 34557167 PMC8453070

[ref10] Fodoulian L. Gschwend O. Huber C. Mutel S. Salazar R. F. Leone R.. (2020). The claustrum-medial prefrontal cortex network controls attentional set-shifting. bioRxiv. doi: 10.1101/2020.10.14.339259

[ref11] Fontané L. Benaiges D. Goday A. Llauradó G. Pedro-Botet J. (2018). Influence of the microbiota and probiotics in obesity. Clin. Investig. Arterioscler. 30, 271–279. doi: 10.1016/j.arteri.2018.03.004 29804899

[ref12] Gainey S. J. Kwakwa K. A. Bray J. K. Pillote M. M. Tir V. L. Towers A. E.. (2016). Short-term high-fat diet (HFD) induced anxiety-like behaviors and cognitive impairment are improved with treatment by glyburide. Front. Behav. Neurosci. 10:156. doi: 10.3389/fnbeh.2016.00156, PMID: 27563288 PMC4980396

[ref13] Harvey P. D. (2019). Domains of cognition and their assessment. Dial. Clin. Neurosci. 21, 227–237. doi: 10.31887/DCNS.2019.21.3/pharvey, PMID: 31749647 PMC6829170

[ref14] Herculano B. Tamura M. Ohba A. Shimatani M. Kutsuna N. Hisatsune T. (2013). β-Alanyl-L-histidine rescues cognitive deficits caused by feeding a high fat diet in a transgenic mouse model of Alzheimer’s disease. J. Alzheimers Dis. 33, 983–997. doi: 10.3233/JAD-2012-121324, PMID: 23099816

[ref15] Hoffman J. D. Yanckello L. M. Chlipala G. Hammond T. C. McCulloch S. D. Parikh I.. (2019). Dietary inulin alters the gut microbiome, enhances systemic metabolism and reduces neuroinflammation in an APOE4 mouse model. PLoS One 14:e0221828. doi: 10.1371/journal.pone.0221828, PMID: 31461505 PMC6713395

[ref16] Huang S. Dong S. Lin L. Ma Q. Xu M. Ni L.. (2023). Inulin ameliorates metabolic syndrome in high-fat diet-fed mice by regulating gut microbiota and bile acid excretion. Front. Pharmacol. 14:14. doi: 10.3389/fphar.2023.1226448, PMID: 37554983 PMC10404850

[ref17] Huang Y. Ying N. Zhao Q. Chen J. Teow S.-Y. Dong W.. (2023). Amelioration of obesity-related disorders in high-fat diet-fed mice following fecal microbiota transplantation from inulin-dosed mice. Molecules 28:3997. doi: 10.3390/molecules28103997, PMID: 37241738 PMC10221449

[ref18] Jangra S. Shekar R. Sharma R. K. Pothuraju R. Mohanty A. K. (2019). Ameliorative effect of fermentable fibres on adiposity and insulin resistance in C57BL/6 mice fed a high-fat and sucrose diet. Food Funct. 10, 3696–3705. doi: 10.1039/c8fo02578a, PMID: 31168538

[ref19] Janssen C. I. F. Jansen D. Mutsaers M. P. C. Dederen P. J. W. C. Geenen B. Mulder M. T.. (2016). The effect of a high-fat diet on brain plasticity, inflammation and cognition in female ApoE4-knockin and ApoE-knockout mice. PLoS One 11:e0155307. doi: 10.1371/journal.pone.0155307, PMID: 27171180 PMC4865084

[ref20] Jones D. T. Graff-Radford J. (2021). Executive dysfunction and the prefrontal cortex. Continuum 27, 1586–1601. doi: 10.1212/CON.0000000000001009 34881727

[ref21] Kadyan S. Park G. Hochuli N. Miller K. Wang B. Nagpal R. (2024). Resistant starches from dietary pulses improve neurocognitive health via gut-microbiome-brain axis in aged mice. Front. Nutr. 11:1322201. doi: 10.3389/fnut.2024.1322201, PMID: 38352704 PMC10864001

[ref22] Kim T.-W. Choi H.-H. Chung Y.-R. (2016). Treadmill exercise alleviates impairment of cognitive function by enhancing hippocampal neuroplasticity in the high-fat diet-induced obese mice. J. Exerc. Rehabil. 12, 156–162. doi: 10.12965/jer.1632644.322, PMID: 27419109 PMC4934958

[ref23] Kothari V. Luo Y. Tornabene T. O’Neill A. M. Greene M. W. Geetha T.. (2017). High fat diet induces brain insulin resistance and cognitive impairment in mice. Biochim Biophys Acta Mol. Basis Dis. 1863, 499–508. doi: 10.1016/j.bbadis.2016.10.006 27771511

[ref24] Lapiz-Bluhm M. D. S. Soto-Piña A. E. Hensler J. G. Morilak D. A. (2009). Chronic intermittent cold stress and serotonin depletion induce deficits of reversal learning in an attentional set-shifting test in rats. Psychopharmacology 202, 329–341. doi: 10.1007/s00213-008-1224-6, PMID: 18587666 PMC2634823

[ref25] Lee S. Kim J. Y. Kim E. Seo K. Kang Y. J. Kim J. Y.. (2018). Assessment of cognitive impairment in a mouse model of high-fat diet-induced metabolic stress with touchscreen-based automated battery system. Exp. Neurobiol. 27, 277–286. doi: 10.5607/en.2018.27.4.277, PMID: 30181690 PMC6120966

[ref26] Leigh S.-J. Morris M. J. (2020). Diet, inflammation and the gut microbiome: mechanisms for obesity-associated cognitive impairment. Biochim Biophys Acta Mol. Basis Dis. 1866:165767. doi: 10.1016/j.bbadis.2020.165767, PMID: 32171891

[ref27] Li X. Chen P. Zhang Y. Zhang J. Shen S. Li K. (2023). Intervention time modified the effect of inulin on high-fat diet-induced obesity and gut microbial disorders. Food Front. 4, 933–944. doi: 10.1002/fft2.243

[ref28] Li D. Song H. Shuo L. Wang L. Xie P. Li W.. (2020). Gonadal white adipose tissue-derived exosomal MiR-222 promotes obesity-associated insulin resistance. Aging 12, 22719–22743. doi: 10.18632/aging.103891, PMID: 33197889 PMC7746358

[ref29] Lin B. Hasegawa Y. Takane K. Koibuchi N. Cao C. Kim-Mitsuyama S. (2016). High-fat-diet intake enhances cerebral amyloid angiopathy and cognitive impairment in a mouse model of Alzheimer’s disease, independently of metabolic disorders. J. Am. Heart Assoc. 5:3154. doi: 10.1161/JAHA.115.003154, PMID: 27412896 PMC4937262

[ref30] Liu Y. Fu X. Lan N. Li S. Zhang J. Wang S.. (2014). Luteolin protects against high fat diet-induced cognitive deficits in obesity mice. Behav. Brain Res. 267, 178–188. doi: 10.1016/j.bbr.2014.02.040, PMID: 24667364

[ref31] Luine V. N. (2014). Estradiol and cognitive function: past, present, and future. Horm. Behav. 66, 602–618. doi: 10.1016/j.yhbeh.2014.08.011, PMID: 25205317 PMC4318702

[ref32] Magnusson K. R. Hauck L. Jeffrey B. M. Elias V. Humphrey A. Nath R.. (2015). Relationships between diet-related changes in the gut microbiome and cognitive flexibility. Neuroscience 300, 128–140. doi: 10.1016/j.neuroscience.2015.05.016, PMID: 25982560

[ref33] Majumdar A. Siva Venkatesh I. P. Basu A. (2023). Short-chain fatty acids in the microbiota–gut–brain axis: role in neurodegenerative disorders and viral infections. ACS Chem. Neurosci. 14, 1045–1062. doi: 10.1021/acschemneuro.2c00803, PMID: 36868874

[ref34] Margolis K. G. Cryan J. F. Mayer E. A. (2021). The microbiota-gut-brain axis: from motility to mood. Gastroenterology 160, 1486–1501. doi: 10.1053/j.gastro.2020.10.066, PMID: 33493503 PMC8634751

[ref35] Markowiak P. Śliżewska K. (2017). Effects of probiotics, prebiotics, and synbiotics on human health. Nutrients 9:1021. doi: 10.3390/nu9091021, PMID: 28914794 PMC5622781

[ref36] Marwitz S. E. Woodie L. N. Blythe S. N. (2015). Western-style diet induces insulin insensitivity and hyperactivity in adolescent male rats. Physiol. Behav. 151, 147–154. doi: 10.1016/j.physbeh.2015.07.023, PMID: 26192711

[ref37] May K. S. den Hartigh L. J. (2021). Modulation of adipocyte metabolism by microbial short-chain fatty acids. Nutrients 13:3666. doi: 10.3390/nu13103666, PMID: 34684670 PMC8538331

[ref38] May K. S. den Hartigh L. J. (2023). Gut microbial-derived short chain fatty acids: impact on adipose tissue physiology. Nutrients 15:272. doi: 10.3390/nu15020272, PMID: 36678142 PMC9865590

[ref39] Mayer E. A. Nance K. Chen S. (2022). The gut-brain axis. Annu. Rev. Med. 73, 439–453. doi: 10.1146/annurev-med-042320-014032 34669431

[ref40] Morrison C. D. Pistell P. J. Ingram D. K. Johnson W. D. Liu Y. Fernandez-Kim S. O.. (2010). High fat diet increases hippocampal oxidative stress and cognitive impairment in aged mice: implications for decreased Nrf2 signaling: high fat diet and brain aging. J. Neurochem. 114, 1581–1589. doi: 10.1111/j.1471-4159.2010.06865.x, PMID: 20557430 PMC2945419

[ref41] O’Riordan K. J. Collins M. K. Moloney G. M. Knox E. G. Aburto M. R. Fülling C.. (2022). Short chain fatty acids: microbial metabolites for gut-brain axis signalling. Mol. Cell Endocrinol. 546:111572. doi: 10.1016/j.mce.2022.111572 35066114

[ref42] Pajkossy P. Szőllősi Á. Demeter G. Racsmány M. (2018). Physiological measures of dopaminergic and noradrenergic activity during attentional set shifting and reversal. Front. Psychol. 9:9. doi: 10.3389/fpsyg.2018.00506, PMID: 29695987 PMC5904264

[ref43] Parks D. H. Tyson G. W. Hugenholtz P. Beiko R. G. (2014). STAMP: statistical analysis of taxonomic and functional profiles. Bioinformatics 30, 3123–3124. doi: 10.1093/bioinformatics/btu494, PMID: 25061070 PMC4609014

[ref44] Pistell P. J. Morrison C. D. Gupta S. Knight A. G. Keller J. N. Ingram D. K.. (2010). Cognitive impairment following high fat diet consumption is associated with brain inflammation. J. Neuroimmunol. 219, 25–32. doi: 10.1016/j.jneuroim.2009.11.010, PMID: 20004026 PMC2823983

[ref45] Qin Y.-Q. Wang L.-Y. Yang X.-Y. Xu Y.-J. Fan G. Fan Y.-G.. (2023). Inulin: properties and health benefits. Food Funct. 14, 2948–2968. doi: 10.1039/d2fo01096h, PMID: 36876591

[ref46] Régnier M. Van Hul M. Roumain M. Paquot A. de Wouters d’Oplinter A. Suriano F.. (2023). Inulin increases the beneficial effects of rhubarb supplementation on high-fat high-sugar diet-induced metabolic disorders in mice: impact on energy expenditure, brown adipose tissue activity, and microbiota. Gut Microbes 15:796. doi: 10.1080/19490976.2023.2178796, PMID: 36803220 PMC9980659

[ref47] Rognes T. Flouri T. Nichols B. Quince C. Mahé F. (2016). VSEARCH: a versatile open source tool for metagenomics. PeerJ 4:e2584. doi: 10.7717/peerj.2584, PMID: 27781170 PMC5075697

[ref48] Romo-Araiza A. Gutiérrez-Salmeán G. Galván E. J. Hernández-Frausto M. Herrera-López G. Romo-Parra H.. (2018). Probiotics and prebiotics as a therapeutic strategy to improve memory in a model of middle-aged rats. Front. Aging Neurosci. 10:10. doi: 10.3389/fnagi.2018.00416, PMID: 30618722 PMC6305305

[ref49] Romo-Araiza A. Picazo-Aguilar R. I. Griego E. Márquez L. A. Galván E. J. Cruz Y.. (2023). Symbiotic supplementation (*E. Faecium* and agave inulin) improves spatial memory and increases plasticity in the hippocampus of obese rats: a proof-of-concept study. Cell Transplant 32:096368972311773. doi: 10.1177/09636897231177357, PMID: 37291807 PMC10272678

[ref50] Sah S. K. Lee C. Jang J.-H. Park G. H. (2017). Effect of high-fat diet on cognitive impairment in triple-transgenic mice model of Alzheimer’s disease. Biochem Biophys Res. Commun. 493, 731–736. doi: 10.1016/j.bbrc.2017.08.122, PMID: 28865961

[ref51] Salinero A. E. Robison L. S. Gannon O. J. Riccio D. Mansour F. Abi-Ghanem C.. (2020). Sex-specific effects of high-fat diet on cognitive impairment in a mouse model of VCID. FASEB J. 34, 15108–15122. doi: 10.1096/fj.202000085R, PMID: 32939871 PMC7737404

[ref52] Schloss P. D. Westcott S. L. Ryabin T. Hall J. R. Hartmann M. Hollister E. B.. (2009). Introducing mothur: open-source, platform-independent, community-supported software for describing and comparing microbial communities. Appl. Environ. Microbiol. 75, 7537–7541. doi: 10.1128/aem.01541-09, PMID: 19801464 PMC2786419

[ref53] Silva Y. P. Bernardi A. Frozza R. L. (2020). The role of short-chain fatty acids from gut microbiota in gut-brain communication. Front. Endocrinol. 11:11. doi: 10.3389/fendo.2020.00025, PMID: 32082260 PMC7005631

[ref54] Snijder M. B. van Dam R. M. Visser M. Seidell J. C. (2006). What aspects of body fat are particularly hazardous and how do we measure them? Int. J. Epidemiol. 35, 83–92. doi: 10.1093/ije/dyi253, PMID: 16339600

[ref55] Spellman T. Svei M. Kaminsky J. Manzano-Nieves G. Liston C. (2021). Prefrontal deep projection neurons enable cognitive flexibility via persistent feedback monitoring. Cell 184, 2750–2766.e17. doi: 10.1016/j.cell.2021.03.047, PMID: 33861951 PMC8684294

[ref56] Szewczyk A. Andres-Mach M. Zagaja M. Kaczmarczyk-Ziemba A. Maj M. Szala-Rycaj J. (2023). The effect of a diet enriched with Jerusalem artichoke, inulin, and fluoxetine on cognitive functions, neurogenesis, and the composition of the intestinal microbiota in mice. Curr. Issues Mol. Biol. 45, 2561–2579. doi: 10.3390/cimb45030168, PMID: 36975538 PMC10047150

[ref57] Tan S. Caparros-Martin J. A. Matthews V. B. Koch H. O’Gara F. Croft K. D.. (2018). Isoquercetin and inulin synergistically modulate the gut microbiome to prevent development of the metabolic syndrome in mice fed a high fat diet. Sci. Rep. 8:10100. doi: 10.1038/s41598-018-28521-8, PMID: 29973701 PMC6031638

[ref58] Tan B. L. Norhaizan M. E. (2019). Effect of high-fat diets on oxidative stress, cellular inflammatory response and cognitive function. Nutrients 11:2579. doi: 10.3390/nu11112579, PMID: 31731503 PMC6893649

[ref59] Thursby E. Juge N. (2017). Introduction to the human gut microbiota. Biochem J. 474, 1823–1836. doi: 10.1042/BCJ20160510, PMID: 28512250 PMC5433529

[ref60] Tooley K. L. (2020). Effects of the human gut microbiota on cognitive performance, brain structure and function: a narrative review. Nutrients 12:3009. doi: 10.3390/nu12103009, PMID: 33007941 PMC7601389

[ref61] Wang Q. Yuan J. Yu Z. Lin L. Jiang Y. Cao Z.. (2018). FGF21 attenuates high-fat diet-induced cognitive impairment via metabolic regulation and anti-inflammation of obese mice. Mol. Neurobiol. 55, 4702–4717. doi: 10.1007/s12035-017-0663-7, PMID: 28712011 PMC5971086

[ref62] Wieërs G. Belkhir L. Enaud R. Leclercq S. Philippart de Foy J.-M. Dequenne I.. (2019). How probiotics affect the microbiota. Front. Cell Infect. Microbiol. 9:454. doi: 10.3389/fcimb.2019.00454, PMID: 32010640 PMC6974441

[ref63] Wu Z. Du Z. Tian Y. Liu M. Zhu K. Zhao Y.. (2022). Inulin accelerates weight loss in obese mice by regulating gut microbiota and serum metabolites. Front. Nutr. 9:9. doi: 10.3389/fnut.2022.980382, PMID: 36245535 PMC9554005

[ref64] Wu Y. Niu X. Li P. Tong T. Wang Q. Zhang M.. (2023). Lactobacillaceae improve cognitive dysfunction via regulating gut microbiota and suppressing Aβ deposits and neuroinflammation in APP/PS1 mice. Arch Microbiol. 205:118. doi: 10.1007/s00203-023-03466-3, PMID: 36928985

[ref65] Zhu L. Qin S. Zhai S. Gao Y. Li L. (2017). Inulin with different degrees of polymerization modulates composition of intestinal microbiota in mice. FEMS Microbiol. Lett. 364:fnx075. doi: 10.1093/femsle/fnx075, PMID: 28407078

[ref66] Zou J. Chassaing B. Singh V. Pellizzon M. Ricci M. Fythe M. D.. (2018). Fiber-mediated nourishment of gut microbiota protects against diet-induced obesity by restoring IL-22-mediated colonic health. Cell Host Microbe 23, 41–53.e4. doi: 10.1016/j.chom.2017.11.003, PMID: 29276170 PMC6005180

[ref67] Zuloaga K. L. Johnson L. A. Roese N. E. Marzulla T. Zhang W. Nie X.. (2016). High fat diet-induced diabetes in mice exacerbates cognitive deficit due to chronic hypoperfusion. J. Cereb Blood Flow Metab 36, 1257–1270. doi: 10.1177/0271678X15616400, PMID: 26661233 PMC4929700

