# Supplementary material for: Supplementation with inulin reverses cognitive flexibility alterations and modulates the gut microbiota in high-fat-fed mice
Source: Front Behav Neurosci. 2024 Nov 6;18:1445154. doi: 10.3389/fnbeh.2024.1445154 (PMC11577567; doi:10.3389/fnbeh.2024.1445154)
Supplement: Supplementary file 1 [file Data_Sheet_1.docx]

***Supplementary Table S1. Effects of fat-added diet and inulin supplementation on AST test performance***

|  |  | **Groups** | | | |  | |
| --- | --- | --- | --- | --- | --- | --- | --- |
| *Stages* |  | **C** | **HF** | **I** | **HFI** | |  |
| *Simple discrimination* |  | 18.0 ± 6.1 | 31.3 ± 7.5 | 27.5 ± 4.5 | 22.5 ± 3.9 | |  |
| *Compound discrimination* |  | 11.9 ± 1.7 | 25.7 ± 5.7 | 10.8 ± 0.9 | 14.5 ± 2.7 | |  |
| *Reversal 1* |  | 27.7 ± 5.4 | 30.0 ± 5.1 | 18.3 ± 2.3 | 24.8 ± 2.9 | |  |
| *Intradimensional shift* |  | 16.3 ± 2.6 | 19.8 ± 3.9 | 17.7 ± 4.0 | 15.0 ± 3.8 | |  |
| *Reversal 2* |  | 25.7 ± 2.5 | 32.7 ± 6.0 | 25.2 ± 4.4 | 22.2 ± 3.4 | |  |
| *Extradimensional shift* |  | 16.9 ± 2.5 | 39.2 ± 6.4 | 22.5 ± 4.4 | 22.2 ± 5.2 | |  |
| *Reversal 3* |  | 20.7 ± 2.1 | 22.3 ± 3.3 | 16.2 ± 1.1 | 19.3 ± 7.0 | |  |

Data are expressed as mean ± standard error. C: Control, HF: High Fat, I: Inulin, HFI: High Fat supplemented with Inulin.

***Supplementary Table S2. Effects of fat-added diet and inulin supplementation on body composition***

|  |  | **Groups** | | | |  | **One-way ANOVA** | | | | |
| --- | --- | --- | --- | --- | --- | --- | --- | --- | --- | --- | --- |
| *Body composition* |  | **C** | **HF** | **I** | **HFI** | | |  | **F _(DF)_** | **P value** |  |
| *Total water (%)* |  | 45.8 ± 2.9 | 49.4 ± 3.0 | 48.0 ± 2.0 | 46.8 ± 4.8 | | |  | 0.2281_(3,21)_ | 0.8758 |  |
| *Extracellular water (%)* |  | 69.7 ± 2.1 | 66.0 ± 4.4 | 72.1 ± 1.2 | 66.7 ± 5.0 | | |  | 0.6491_(3,21)_ | 0.5923 |  |
| *Intracellular water (%)* |  | 30.8 ± 2.0 | 34.0 ± 4.4 | 27.9 ± 1.2 | 33.3 ± 4.9 | | |  | 0.6292_(3,21)_ | 0.6043 |  |
| *Free fat mass (%)* |  | 62.5 ± 4.0 | 67.5 ± 4.1 | 65.6 ± 2.7 | 64.0 ± 6.5 | | |  | 0.2282_(3,21)_ | 0.8757 |  |
| *Fat mass (%)* |  | 37.5 ± 4.0 | 32.5 ± 4.1 | 34.4 ± 2.7 | 36.0 ± 6.5 | | |  | 0.2282_(3,21)_ | 0.8757 |  |
| *BMI (kg/m^2^)* |  | 6.4 ± 0.2 | 6.5 ± 0.4 | 5.8 ± 0.2 | 6.1 ± 0.3 | | |  | 1.156_(3,21)_ | 0.3499 |  |

Data are expressed as mean ± standard error. C: Control, HF: High Fat, I: Inulin, HFI: High Fat supplemented with Inulin, BMI: Body-mas Index.

***Supplementary Table S3. Effects of fat-added diet and inulin supplementation on fat distribution***

|  |  | **Groups** | | | |  | **One-way ANOVA** | | | | |
| --- | --- | --- | --- | --- | --- | --- | --- | --- | --- | --- | --- |
| *Adipose tissue* |  | **C** | **HF** | **I** | **HFI** | | |  | **F _(DF)_** | **P value** |  |
| *Inguinal (%)* |  | 0.79 ± 0.21 | 1.02 ± 0.14 | 0.64 ± 0.12 | 0.78 ± 0.15 | | |  | 0.8558_(3,21)_ | 0.4792 |  |
| *Gonadal (%)* |  | 1.03 ± 0.24 | 2.04 ± 0.35 | 1.07 ± 0.22 | 1.37 ± 0.21 | | |  | 3.237_(3,21)_ | 0.0428 |  |
| *Retroperitoneal (%)* |  | 0.25 ± 0.07 | 0.80 ± 0.18 | 0.58 ± 0.30 | 0.57 ± 0.05 | | |  | 1.826_(3,21)_ | 0.1734 |  |
| *Peritoneal (%)* |  | 1.85 ± 0.14 | 2.80 ± 0.11 | 1.81 ± 0.29 | 2.51 ± 0.20 | | |  | 6.105_(3,21)_ | 0.0037 |  |

Data are expressed as mean ± standard error. C: Control, HF: High Fat, I: Inulin, HFI: High Fat supplemented with Inulin.

***Supplementary Table S4. Tukey´s multiple comparisons test on fat distribution analysis***

|  | | **Gonadal adipose tissue** | | |  | | **Peritoneal adipose tissue** | | | |
| --- | --- | --- | --- | --- | --- | --- | --- | --- | --- | --- |
| *Group comparisons* | **P value** | | **Summary** |  | | **P value** | | **Summary** | |  |
| *Control vs High fat* | 0.0481 | | * |  | | 0.0121 | | * |  |  |
| *Control vs Inulin* | 0.9997 | | ns |  | | 0.9986 | | ns |  |  |
| *Control vs High fat/Inulin* | 0.7792 | | ns |  | | 0.1083 | | ns |  |  |
| *High fat vs Inulin* | 0.0712 | | ns |  | | 0.0115 | | * |  |  |
| *High fat vs High fat/Inulin* | 0.3056 | | ns |  | | 0.7485 | | ns |  |  |
| *Inulin vs High fat/Inulin* | 0.8427 | | ns |  | | 0.0960 | | ns |  |  |

Data are expressed as mean ± standard error; * p < 0.05, ns: no significative.
